# Socioeconomic inequality in domains of health: results from the World Health Surveys

**DOI:** 10.1186/1471-2458-12-198

**Published:** 2012-03-19

**Authors:** Ahmad Reza Hosseinpoor, Jennifer Anne Stewart Williams, Lynn Itani, Somnath Chatterji

**Affiliations:** 1 Department of Health Statistics and Information Systems, World Health Organization, Geneva, Switzerland; 2 Research Centre for Gender, Health & Ageing, University of Newcastle, Australia; 3 Institute for Development, Research, Advocacy and Applied Care, Beirut, Lebanon

## Abstract

**Background:**

In all countries people of lower socioeconomic status evaluate their health more poorly. Yet in reporting overall health, individuals consider multiple domains that comprise their perceived health state. Considered alone, overall measures of self-reported health mask differences in the domains of health. The aim of this study is to compare and assess socioeconomic inequalities in each of the individual health domains and in a separate measure of overall health.

**Methods:**

Data on 247,037 adults aged 18 or older were analyzed from 57 countries, drawn from all national income groups, participating in the World Health Survey 2002-2004. The analysis was repeated for lower- and higher-income countries. Prevalence estimates of poor self-rated health (SRH) were calculated for each domain and for overall health according to wealth quintiles and education levels. Relative socioeconomic inequalities in SRH were measured for each of the eight health domains and for overall health, according to wealth quintiles and education levels, using the relative index of inequality (RII). A RII value greater than one indicated greater prevalence of self-reported poor health among populations of lower socioeconomic status, called pro-rich inequality.

**Results:**

There was a descending gradient in the prevalence of poor health, moving from the poorest wealth quintile to the richest, and moving from the lowest to the highest educated groups. Inequalities which favor groups who are advantaged either with respect to wealth or education, were consistently statistically significant in each of the individual domains of health, and in health overall. However the size of these inequalities differed between health domains. The prevalence of reporting poor health was higher in the lower-income country group. Relative socioeconomic inequalities in the health domains and overall health were higher in the higher-income country group than the lower-income country group.

**Conclusions:**

Using a common measurement approach, inequalities in health, favoring the rich and the educated, were evident in overall health as well as in every health domain. Existent differences in averages and inequalities in health domains suggest that monitoring should not be limited only to overall health. This study carries important messages for policy-making in regard to tackling inequalities in specific domains of health. Targeting interventions towards individual domains of health such as mobility, self-care and vision, ought to be considered besides improving overall health.

## Background

Inequities in health constitute one of the main challenges for public health globally. In all countries people of lower socioeconomic status (SES), as measured by social determinants such as education, income or occupation, are in a worse state of health compared to those from higher SES across the entire range. Health is defined by the World Health Organization (WHO) as "a state of complete physical, mental and social well-being and not merely the absence of disease or infirmity" [1]. However, for the purposes of measurement, health must be operationalized as an individual's intrinsic capacity to function in a range of domains and aggregated in order to quantify and compare levels of health across individuals and populations. Uniform approaches to measuring levels and distributions of health allow targeted approaches by policy makers to tackle health inequalities and researchers to monitor the impact of these interventions [2-7].

Self-rated health (SRH) [8,9] is widely used in population surveys to measure health status. Empirically SRH is a strong predictor of mortality risk, even after accounting for known socioeconomic and medical risk factors [10-14]. Responses to a single-item question, such as, "how would you rate your health in general" summarize an individual's overall self-report of their health combining the individual's aggregation of her functioning without a way of determining the relative problems in different domains [10,15]. However for monitoring population health over time, and particularly for analyses of socioeconomic disparities, the overall SRH question may create comparability problems [16].

A common theme that has emerged from efforts to develop an operational definition of "health" is the view that health state reported by individuals consists of a series of values indicating levels on domains such as mobility, pain, sleep, cognition and vision [2]. While overall measures of SRH are important, understanding the key components of health can help inform policies and interventions to improve the different aspects of health and health outcomes in general.

In recent decades the measurement of health and its core elements has been operationalized through a set of elements, or domains, that together constitute an understanding of overall health [17]. The WHO developed eight core domains of health that have been widely accepted as being of fundamental importance to all human beings irrespective of their social or socioeconomic circumstances. These domains are: mobility; self-care; pain and discomfort; cognition; interpersonal activities; vision; sleep and energy, and affect [2,17]. Questions referring to the domains of health are used in many population survey instruments. For measurement purposes self-reported health in each domain is characterized by a single cardinal scale [2]. Although there are methodological challenges pertaining to the comparability of health status data across populations and cultures, research has shown that the WHO health domains are highly consistent across countries and cultures [18]. The availability of multi-domain health state measurement instruments makes it possible to measure the different attributes of health in population surveys conducted across many countries [2,19,20].

A few studies have demonstrated associations between SES and physical, functional, mental, cognitive and behavioral aspects of health [9,21-24]. However mostly these studies focus only on specific sub-populations in high income countries so the results are often not generalizable. We know of no work that has comprehensively "un-packed" socioeconomic associations separately within the WHO domains of health at a multi-country level. The aim of this study is to compare and assess socioeconomic inequalities in each of the individual health domains and in a separate measure of overall health.

## Methods

### Study population

The study population comprises a multi-country dataset drawn from the World Health Survey (WHS). The WHS was conducted by the World Health Organization in 2002-2004 to provide valid, reliable, representative and comparable population data on the health status of adults, aged 18 years and older, in 70 countries from all regions of the world [25]. All samples were probabilistically selected with every individual being assigned a known non-zero probability of being selected. The samples were nationally representative except in China, Comoros, Congo, Côte d'Ivoire, India, and the Russian Federation, where the WHS was carried out in geographically limited regions. To adjust for the population distribution represented by the UN Statistical Division http://unstats.un.org/unsd/default.htm and also non-response, post-stratification corrections were made to sampling weights [26].

### Data

This study includes 57 countries drawn from all national income groups as defined by the World Bank [27]. Thirty seven low- and lower middle-income countries and twenty upper middle- and high-income countries were further combined into the lower-income and the higher-income country groups, respectively. Inclusion at the country level required complete information on sampling weights and key covariates. Eleven of the thirteen excluded countries had insufficient data on sampling weights and two had insufficient data to create the household wealth index. The study included 247,037 respondents, aged 18 and over. Appendix table 1 gives the sample size by country. Separate analyses were undertaken for pooled data sets of 57 countries, 37 lower-income countries, and 20 higher-income countries. The data used here are openly available.

**Table 1:** Prevalence of self-reported poor health across health domains and overall health among adults aged 18+, by wealth and education; pooled analysis of 57 countries WHS (2002-2004)

	Overall health		Mobility		Self-care		Pain and discomfort		Cognition		Interpersonal activities		Vision		Sleep and Energy		Affect	
	**%**	**SE**	**%**	**SE**	**%**	**SE**	**%**	**SE**	**%**	**SE**	**%**	**SE**	**%**	**SE**	**%**	**SE**	**%**	**SE**

**Average**	**9.4**	0.2	**6.2**	0.1	**2.9**	0.1	**10.8**	0.2	**6.2**	0.1	**3.2**	0.1	**4.8**	0.1	**7.6**	0.2	**7.8**	0.2

**Wealth quintile 1**	**13.9**	0.4	**9.2**	0.4	**4.5**	0.3	**14.3**	0.5	**8.7**	0.3	**4.5**	0.2	**6.9**	0.3	**9.7**	0.4	**10.1**	0.4

**Wealth quintile 2**	**11.2**	0.4	**6.9**	0.3	**3.4**	0.2	**12.0**	0.4	**6.9**	0.3	**3.7**	0.2	**5.7**	0.2	**8.6**	0.3	**8.5**	0.3

**Wealth quintile 3**	**9.5**	0.4	**6.1**	0.3	**2.7**	0.2	**11.3**	0.4	**6.3**	0.3	**3.1**	0.2	**4.8**	0.2	**7.9**	0.3	**8.0**	0.3

**Wealth quintile 4**	**7.7**	0.3	**5.1**	0.2	**2.6**	0.2	**9.7**	0.3	**5.6**	0.3	**2.9**	0.2	**3.7**	0.2	**6.6**	0.3	**7.4**	0.3

**Wealth quintile 5**	**5.3**	0.2	**4.1**	0.2	**1.7**	0.1	**7.4**	0.3	**3.9**	0.2	**2.0**	0.1	**3.2**	0.2	**5.7**	0.3	**5.5**	0.2

**No formal education**	**14.5**	0.4	**10.3**	0.3	**5.6**	0.2	**16.3**	0.5	**10.4**	0.4	**5.6**	0.3	**8.3**	0.3	**10.6**	0.4	**11.1**	0.4

**Less than primary schooling**	**12.2**	0.5	**7.9**	0.4	**3.7**	0.3	**15.3**	0.5	**8.9**	0.4	**4.4**	0.3	**6.7**	0.3	**10.3**	0.4	**10.1**	0.4

**Primary school completed**	**8.5**	0.3	**5.4**	0.3	**2.7**	0.2	**10.8**	0.4	**5.9**	0.2	**3.1**	0.2	**4.0**	0.2	**7.8**	0.3	**8.5**	0.3

**Secondary school completed**	**6.8**	0.3	**4.2**	0.2	**1.6**	0.1	**7.1**	0.3	**4.1**	0.2	**1.7**	0.1	**3.1**	0.2	**5.2**	0.2	**5.5**	0.2

**High school completed**	**5.8**	0.3	**3.4**	0.2	**1.2**	0.1	**6.5**	0.3	**2.7**	0.2	**1.6**	0.1	**2.4**	0.1	**5.2**	0.3	**5.0**	0.2

### Variables

Respondents to the WHS Individual Questionnaire were asked to self-rate the extent to which they were having difficulties in each of eight health domains using a five point scale: none; mild; moderate; severe, and extreme/cannot do. In addition respondents were asked to rate their overall health as either: very good; good; moderate; bad, or very bad.

The outcome variable in this study is the dichotomy poor health (comprising the health domain responses "severe" and "extreme/cannot do" and the overall health responses "bad" or "very bad") vs. good health (comprising the health domain responses "none", "mild" or "moderate" and the overall health responses "very good", "good" and "moderate"). This binary outcome variable was calculated for each health domain and for overall health.

Education and wealth are independent variables used here as categorical measures of SES. For education, individuals were assigned an educational ranking that was either: no formal schooling; less than primary school; completion of primary school; completion of secondary school, or completion of high school or above. For wealth, a dichotomous hierarchical ordered probit model was used to develop an index of the household economic status or wealth. This indicated individuals' economic status and was based on owning selected assets and/or with access to certain services [28-30]. The index was divided into quintiles within each country, where quintile one represented the poorest wealth quintile and quintile five the richest.

### Methods of analysis

Prevalence estimates of poor health were calculated for each domain of health and for overall health (overall prevalence) according to wealth quintiles and education level. In addition, relative socioeconomic inequalities in SRH were measured for each of the eight health domains and for overall health using the relative index of inequality (RII). The RII summarized the extent to which perceived poor health varied separately by education and wealth in each of the health domains, by taking into account the distribution of poor health as well as the distribution of the population according to our measures of education and wealth [31]. To calculate RII, individuals were cumulatively ranked (ranging from zero to one) according to descending socioeconomic status (i.e. highest wealth or education level to lowest). The RII represents the ratio reporting poor health between those at the top rank (i.e. the lowest level of education or wealth) and those at rank zero (i.e. the highest level of education or wealth). Thus RII > 1 indicates a higher proportion rating poor health among populations of lower socioeconomic status. We refer to this situation as *pro-rich *inequality; *pro-poor *inequality exists when the prevalence of poor health is higher among those with higher socioeconomic status [32].

Data were adjusted for respondents' country of residence and age (expressed categorically as 18-29, 30-39, 40-49, 50-59, 60-69 and 70+ years) in Model 1. In Model 2 adjustments were also made for: sex; urban/rural area of residence; marital status (married/cohabiting vs. never married vs. divorced/separated/widowed), and wealth or education as possible confounders.

The Poisson regression model with a robust variance was used to assess associations in each health domain and in overall health and to generate prevalence ratio values and 95% confidence intervals (CIs) [33].

All analyses were weighted to account for individual survey sample designs and adjustments were made to allow for the fact that observations within survey clusters were not necessarily independent. Analyses were carried out using STATA V11 (StataCorp, 2009).

## Results

### Prevalence of reporting poor health

Table 1 gives prevalence of poor health across the health domains and overall health, by wealth and education for all countries. Tables 2 and 3 give prevalence estimates for the lower- and higher-income country groups respectively. On average, the prevalence of poor health was lowest for self-care (2.9% for all countries, 3.4% for the lower-income country group, and 1.7% for the higher-income country group) and highest for pain and discomfort (10.8% for all countries, 11.2% for the lower-income country group, and 9.7% for the higher-income country group). The prevalence of reporting poor overall health was 9.4% for all countries, 10.1% for the lower-income country group and 7.2% for the higher-income country group.

**Table 2:** Prevalence of self-reported poor health across health domains and overall health among adults aged 18+, by wealth and education; pooled analysis of 37 lower-income countries WHS (2002-2004)

	Overall health		Mobility		Self-care		Pain and discomfort		Cognition		Interpersonal activities		Vision		Sleep and Energy		Affect	
	**%**	**SE**	**%**	**SE**	**%**	**SE**	**%**	**SE**	**%**	**SE**	**%**	**SE**	**%**	**SE**	**%**	**SE**	**%**	**SE**

**Average**	**10.1**	0.3	**6.7**	0.2	**3.4**	0.1	**11.2**	0.3	**6.5**	0.2	**3.6**	0.1	**5.1**	0.1	**7.5**	0.2	**7.8**	0.2

**Wealth quintile 1**	**14.6**	0.5	**9.6**	0.4	**5.0**	0.3	**14.6**	0.5	**8.9**	0.4	**5.0**	0.3	**7.0**	0.3	**9.8**	0.4	**10.1**	0.4

**Wealth quintile 2**	**11.9**	0.4	**7.2**	0.3	**3.8**	0.2	**12.3**	0.5	**7.0**	0.3	**4.0**	0.2	**5.9**	0.3	**8.6**	0.4	**8.3**	0.3

**Wealth quintile 3**	**10.2**	0.4	**6.9**	0.4	**3.1**	0.2	**11.6**	0.4	**6.5**	0.3	**3.3**	0.2	**5.3**	0.3	**7.4**	0.3	**7.9**	0.3

**Wealth quintile 4**	**8.5**	0.4	**5.7**	0.3	**3.1**	0.2	**10.1**	0.4	**6.1**	0.4	**3.4**	0.3	**4.0**	0.2	**6.7**	0.3	**7.5**	0.4

**Wealth quintile 5**	**6.1**	0.3	**4.7**	0.3	**2.1**	0.2	**8.0**	0.4	**4.4**	0.2	**2.4**	0.2	**3.8**	0.2	**5.5**	0.3	**5.5**	0.3

**No formal education**	**13.9**	0.4	**9.9**	0.4	**5.5**	0.2	**15.8**	0.5	**9.8**	0.4	**5.5**	0.3	**8.0**	0.3	**9.9**	0.4	**10.5**	0.4

**Less than primary schooling**	**11.7**	0.5	**7.9**	0.5	**3.8**	0.3	**14.1**	0.6	**7.9**	0.4	**4.2**	0.3	**6.3**	0.4	**9.5**	0.4	**9.1**	0.4

**Primary school completed**	**8.2**	0.4	**5.0**	0.3	**2.6**	0.2	**9.6**	0.4	**5.3**	0.3	**3.1**	0.2	**3.5**	0.2	**6.9**	0.3	**7.5**	0.3

**Secondary school completed**	**7.4**	0.4	**4.6**	0.3	**2.0**	0.2	**6.7**	0.4	**4.7**	0.3	**2.1**	0.2	**3.4**	0.4	**5.2**	0.3	**5.2**	0.3

**High school completed**	**7.5**	0.4	**4.3**	0.3	**1.6**	0.2	**7.5**	0.4	**3.1**	0.2	**1.9**	0.2	**3.0**	0.2	**5.1**	0.3	**5.2**	0.3

**Table 3:** Prevalence of self-reported poor health across health domains and overall health among adults aged 18+, by wealth and education; pooled analysis of 20 higher-income countries WHS (2002-2004)

	Overall health		Mobility		Self-care		Pain and discomfort		Cognition		Interpersonal activities		Vision		Sleep and Energy		Affect	
	**%**	**SE**	**%**	**SE**	**%**	**SE**	**%**	**SE**	**%**	**SE**	**%**	**SE**	**%**	**SE**	**%**	**SE**	**%**	**SE**

**Average**	**7.2**	0.3	**4.6**	**0.2**	**1.7**	0.1	**9.7**	0.3	**5.3**	0.2	**2.1**	0.1	**3.7**	0.2	**7.8**	0.3	**7.9**	0.3

**Wealth quintile 1**	**12.0**	0.8	**7.9**	**0.8**	**3.1**	0.4	**13.5**	0.9	**8.0**	0.6	**3.0**	0.4	**6.5**	0.6	**9.4**	0.7	**10.0**	0.7

**Wealth quintile 2**	**9.4**	0.6	**6.1**	**0.5**	**2.2**	0.3	**11.2**	0.7	**6.8**	0.6	**2.7**	0.3	**5.0**	0.5	**8.8**	0.6	**9.0**	0.6

**Wealth quintile 3**	**7.5**	0.6	**4.0**	**0.3**	**1.6**	0.3	**10.4**	0.6	**5.7**	0.5	**2.5**	0.3	**3.1**	0.3	**9.3**	0.6	**8.5**	0.6

**Wealth quintile 4**	**5.4**	0.4	**3.4**	**0.4**	**1.1**	0.2	**8.6**	0.6	**4.2**	0.4	**1.7**	0.3	**2.9**	0.3	**6.3**	0.5	**7.3**	0.5

**Wealth quintile 5**	**3.4**	0.4	**2.6**	**0.3**	**0.9**	0.2	**5.9**	0.5	**2.8**	0.3	**1.2**	0.2	**1.8**	0.2	**6.0**	0.6	**5.5**	0.5

**No formal education**	**25.7**	1.5	**17.2**	**1.4**	**6.4**	0.9	**24.7**	1.6	**21.5**	1.7	**7.0**	1.1	**15.1**	1.4	**22.2**	1.5	**22.0**	1.6

**Less than primary schooling**	**14.4**	1.0	**8.1**	**0.8**	**3.4**	0.5	**20.5**	1.4	**13.1**	1.0	**5.1**	0.7	**8.1**	0.8	**13.8**	1.0	**14.3**	1.1

**Primary school completed**	**9.2**	0.7	**6.6**	**0.7**	**2.8**	0.5	**13.9**	0.8	**7.5**	0.5	**2.8**	0.3	**5.4**	0.6	**10.2**	0.6	**11.2**	0.7

**Secondary school completed**	**5.9**	0.4	**3.5**	**0.3**	**0.9**	0.1	**7.8**	0.5	**3.3**	0.3	**1.2**	0.1	**2.4**	0.2	**5.3**	0.3	**6.0**	0.3

**High school completed**	**3.4**	0.3	**2.1**	**0.2**	**0.7**	0.1	**4.8**	0.3	**2.1**	0.2	**1.2**	0.1	**1.4**	0.1	**5.4**	0.4	**4.6**	0.3

### Wealth-related inequality in poor health

Poor health was more prevalent in the poorest wealth quintile than in the richest. There was a descending gradient in the prevalence of poor health, moving from the poorest wealth quintile to richest, in all the domains of health and in overall health.

**Figure 1:**
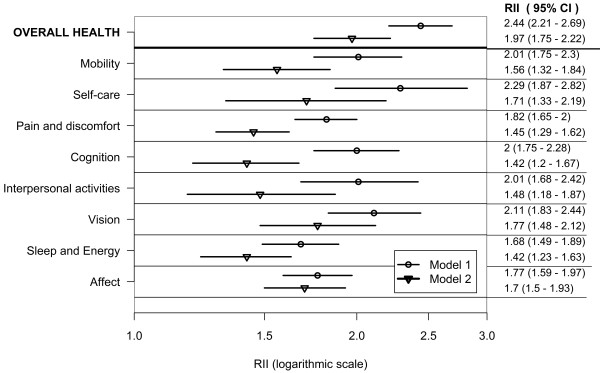
**Wealth-related inequality in poor health by health domains and overall health in adults aged 18+; pooled analysis of 57 countries WHS (2002-2004)**.

**Figure 2:**
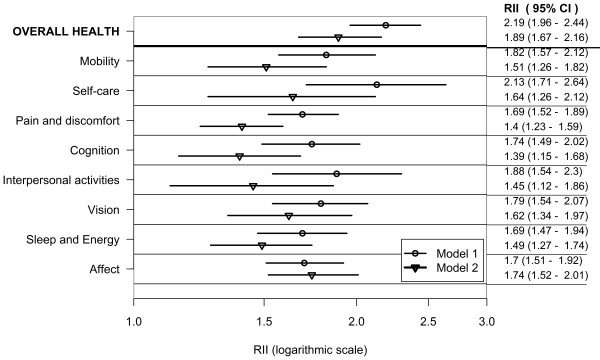
**Wealth-related inequality in poor health by health domains and overall health in adults aged 18+; pooled analysis of 37 lower-income countries WHS (2002-2004)**.

**Figure 3:**
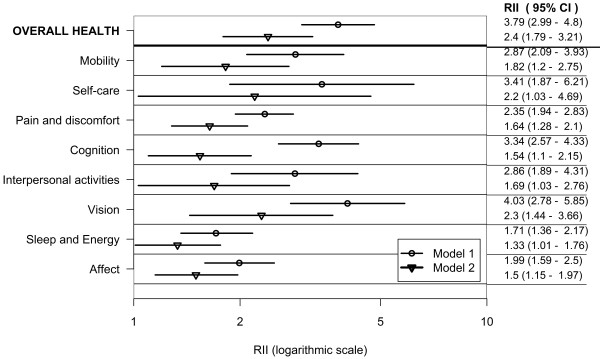
**Wealth-related inequality in poor health by health domains and overall health in adults aged 18+; pooled analysis of 20 higher-income countries WHS (2002-2004)**.

Figures 1, 2 and 3 show wealth-related relative inequalities, in poor health in the domains of health, and overall health, for all countries, the lower-income country group, and the higher-income country group respectively. In each of the country groups, the RII was greater than one and statistically significant in all domains and in overall health indicating pro-rich inequalities. Model 1 was adjusted for age and country of residence. Inequalities attenuated after controlling for age, sex, education, marital status, place and country of residence in Model 2.

In the combined countries data set poor health was twice as prevalent in the poorest, compared with the richest adults in the mobility, self-care, cognition, interpersonal activities and vision domains in Model 1. The prevalence of poor overall health was twice as high in the richest, compared with the poorest wealth quintile, in both Models 1 and 2.

In the lower-income country group (Model 1) poor health was twice as prevalent in the poorest, compared with the richest adults in the self-care domain and in overall health. In the higher-income country group (Model 1) poor health was at least twice as prevalent in the poorest compared with the richest adults in all the domains except sleep and energy.

### Education-related inequality in poor health

Poor health was more prevalent in the lowest compared with the highest education level. There was a descending gradient in the prevalence of poor health, moving from the lowest to the highest education level, in all the domains of health and in overall health.

**Figure 4:**
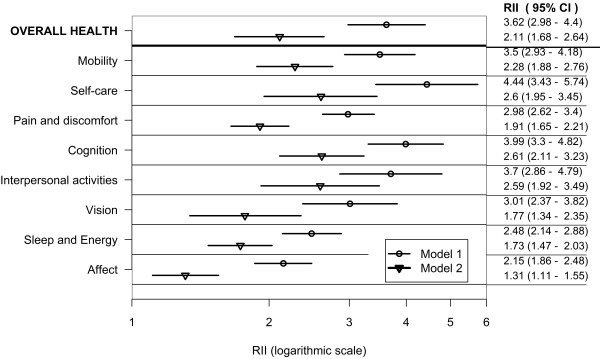
**Education-related inequality in poor health by health domains and overall health in adults aged 18+; pooled analysis of 57 countries WHS (2002-2004)**.

**Figure 5:**
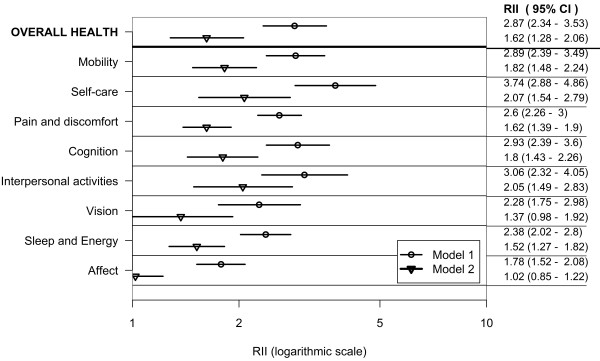
**Education-related inequality in poor health by health domains and overall health in adults aged 18+; pooled analysis of 37 lower-income countries WHS (2002-2004)**.

**Figure 6:**
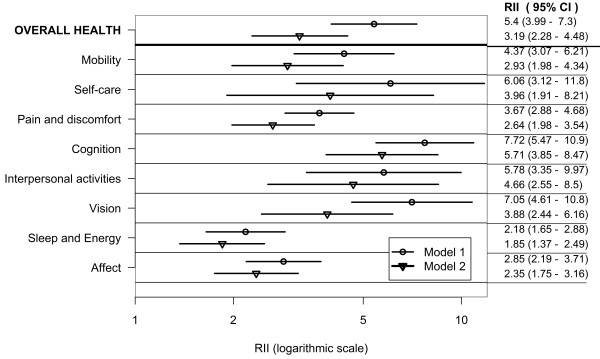
**Education-related inequality in poor health by health domains and overall health in adults aged 18+; pooled analysis of 20 higher-income countries WHS (2002-2004)**.

Figures 4, 5 and 6 show education-related relative inequalities, in poor health in the domains of health, and overall health for all countries, the lower-income country group, and the higher-income country group, respectively. For each country group, the RII was greater than one and statistically significant in all domains and in overall health indicating pro-rich inequalities (Model 1). Inequalities attenuated after controlling for age, sex, wealth, marital status, place and country of residence in Model 2. The RII was not significant in the vision and affect domains in Model 2 for the lower-income country group.

In the combined countries data set (Model 1) poor health was at least three times as prevalent in adults in the lowest, compared with the highest education rank in the mobility, self-care, pain and discomfort, cognition, interpersonal activities and vision domains. Poor overall health was over three times as prevalent in the lowest, compared with the highest education rank in Model 1 and twice as high in Model 2.

In the lower-income country group (Model 1) poor health was at least three times as prevalent in adults in the lowest, compared with the highest education rank in the self-care, and interpersonal activities domains. In the higher-income country group (Model 1) poor health was at least three times as prevalent in adults in the lowest, compared with the highest education rank in all of the health domains except affect and sleep and energy.

## Discussion

Pro-rich health inequalities, which favor groups who are advantaged either with respect to education or wealth, are consistent and statistically significant in each of the domains of health and in health overall. There was a descending gradient in the prevalence of poor health, moving from the poorest wealth quintile to richest, and moving from the lowest to the highest educated groups.

In the combined countries data set, in all the domains and in overall health, the education-related inequality in poor health was higher than the wealth-related inequality in poor health. Adjusting for the effects of sex, urban/rural area, marital status and education or wealth as other possible confounders on top of age and country of residence attenuated our measured inequalities, but all of the inequalities remained statistically significant. These findings are consistent with the literature reporting positive associations between SES (measured by education, income and wealth) and SRH [8,34].

Reports of health status incorporate complex combinations of an individual's assessment about their health and health conditions [6]. Considered alone, summary measures of overall health mask differences in the domains of health that may be important to know about in order to target specific interventions. This study takes the additional step of assessing socioeconomic inequalities in the individual domains of health and comparing these measures with inequalities in overall health measured separately. The findings contribute to current evidence of health inequalities by reporting statistically significant inequalities in poor health separately within the widely used domains of health developed by the WHO.

In the combined countries data set and for both country income groups, inequalities were highest for self-care, cognition, vision and mobility. It is possible that correlations between the domains to some extent influence these results, for example persons reporting poor self-care may also report poor mobility and poor cognition. Sadana et al. [17] assessed correlations between six WHO health domains (affect, cognition, mobility, pain, self-care and usual activities) and overall health from 66 surveys carried out by the WHO and showed relatively high correlations between self-care and mobility and self-care and cognition (Rho: 0.76 and 0.59, respectively).

Associations between SES and aspects of SRH [22,35,36] are widely documented. The ageing of populations has increased interest in cognitive function as a public health issue. There is now a mounting body of evidence that low SES, measured by education, occupation, income, and ownership of financial assets, predicts decline in cognitive function in older adults [37-41].

Internationally there is a recognized need to address the "social determinants of health" [4,42,43]. Yet taking action to reduce inequalities and inequities in health within countries requires understanding of how social and economic factors are associated with all of the key components of health, as well as health overall. This is the first study of its kind to provide evidence of associations between education and wealth specifically in all eight domains of health at a pooled multi-country level, and also by comparing groups of lower- and higher-income countries. The analysis by country income groups provides additional insights into the patterning of relative socioeconomic inequalities in the domains of health. Although, on average, health is better in the higher-income countries, the distribution of individual health states in accordance with educational rank as well as wealth rank is more unequal in this group.

### Study strengths

The large multi-country dataset allowed us to assess socioeconomic inequalities in the health domains and in overall health. We have ensured comparability of data between countries by using the WHS. Inequalities in health are measured here by SRH. Self-rated health instruments are applicable within many cultural, demographic and socioeconomic settings [10,44-46] and are strong predictors of health care utilization, morbidity and mortality outcomes [12-14,16,34,47].

This work is based on the domains of health developed by the WHO. They provide a consistent validated way of describing and comparing population health within and between countries [2,17].

Higher SES is associated with better living standards, the most direct (and popular) measures of which are income and consumption expenditure. However measuring income and consumption expenditure can be problematic. In high-income countries, consumption expenditure patterns are very complex and income can be a better measure, and in developing countries, where formal employment is less common and home production widespread, consumption expenditure can be a more accurate measure of living standards [48]. This study uses a method to estimate household wealth that is based on the premise that wealthier households are more likely to own a given set of assets. In addition to being consistent with a broader definition of poverty, this approach provides a way of drawing international comparisons when analyzing health inequalities [28-30].

It is important to consider evidence of socioeconomic inequalities when developing interventions that target specific domains (e.g. mobility, pain and cognition), otherwise interventions may widen inequalities in health. The results from this study are of relevance to public health policy-makers and others because they identify inequalities in the individual health domains.

### Limitations

The data are cross sectional and so can only describe associations between socioeconomic factors and health. The results show that health inequalities according to education and wealth exist after adjusting for age, at a point in time, but they do not explain casual relationships or health change over time. There is a need for research that examines ways in which socioeconomic factors mediate changes in health domains as well as overall health.

The results of this study are based on self-reported information about health. Future studies need to incorporate health examinations and biomarkers within household surveys in order to improve the validity of self-reported health states and to detect and correct systematic reporting biases.

The countries were not probabilistically selected and therefore not necessarily representative of the world or of similar groups of countries (e.g. defined by geography or income). The use of pooled data masks possibly important variation between countries. For example, in order to tailor intervention programs aimed at improving health it is important to understand SRH within local context and culture and these aspects are not captured in our data [44]. Although it was not the purpose of this study to examine inter-country variation, we did include a "country" variable to control for any potential confounding effects related to individual countries.

There is evidence that relative to advantaged groups, disadvantaged groups may fail to perceive and report the presence of illness or health deficits which may result in misleading assessments of population health [9,34,49]. However if such bias exists in our study, then it is likely that the results underestimate the true size of education-and wealth-related inequalities in the domains of health and health overall.

Lastly, we used RII which is a relative measure of inequality that is adjusted for variation in average prevalence of poor health across health domains. As such, information on the absolute size of differences in prevalence of poor health between socioeconomic groups is not reflected in RII [50]. To address this we have provided prevalence estimates of poor health by wealth quintile and educational level.

Deconstructing inequalities in health in different domains, separately by wealth and education in lower- and higher-income country groups, paints a much more nuanced picture than would be otherwise visible. Major differences between the country groups highlight the complex interaction between health, a country's level of economic development and individual socioeconomic status.

## Conclusions

In order to understand drivers of inequality in the distribution of health status within country settings, one needs to examine not just overall self-reported health (as the inequality in this overall item does not change much by wealth or education) but the individual domains of health status and disentangle them by different SES stratifiers. Future studies should attempt to improve our understanding of the drivers of these varying patterns. This will then provide the evidence required for specific areas to be addressed in targeted population sub-groups.

## Competing interests

The authors declare that they have no competing interests.

## Authors' contributions

AH designed the study with input from SC, JSW and LI. LI conducted the literature review. AH did the statistical analysis. JSW wrote the first draft with inputs from AH and SC. All co-authors read and approved the final draft.

## Pre-publication history

The pre-publication history for this paper can be accessed here:

http://www.biomedcentral.com/1471-2458/12/198/prepub
